# Synthesis of transfer-free graphene on cemented carbide surface

**DOI:** 10.1038/s41598-018-23206-8

**Published:** 2018-03-19

**Authors:** Xiang Yu, Zhen Zhang, Fei Liu, Yi Ren

**Affiliations:** 1 Beijing Key Laboratory of Materials Utilisation of Nonmetallic Minerals and Solid Wastes, National Laboratory of Mineral Materials, School of Materials Science and Technology, China University of Geosciences (Beijing), Beijing, 100083 PR China; 2 School of Engineering and Technology, China University of Geosciences (Beijing), Beijing, 100083 PR China

## Abstract

Direct growth of spherical graphene with large surface area is important for various applications in sensor technology. However, the preparation of transfer-free graphene on different substrates is still a challenge. This study presents a novel approach for the transfer-free graphene growth directly on cemented carbide. The used simple thermal annealing induces an in-situ transformation of magnetron-sputtered amorphous silicon carbide films into the graphene matrix. The study reveals the role of Co, a binding phase in cemented carbides, in Si sublimation process, and its interplay with the annealing temperature in development of the graphene matrix. A detailed physico-chemical characterisation was performed by structural (XRD analysis and Raman spectroscopy with mapping studies), morphological (SEM) and chemical (EDS) analyses. The optimal bilayer graphene matrix with hollow graphene spheres on top readily grows at 1000 °C. Higher annealing temperature critically decreases the amount of Si, which yields an increased number of the graphene layers and formation of multi-layer graphene (MLG). The proposed action mechanism involves silicidation of Co during thermal treatment, which influences the existing chemical form of Co, and thus, the graphene formation and variations in a number of the formed graphene layers.

## Introduction

Synthesis of graphene on different substrates is a challenging and attractive approach that can broaden application spectra of currently existing engineering materials. Versatile experimental approaches were used, but major constraints are related to synthesis of adequate graphene sheets and subsequent application problems caused by the transfer of synthesised graphene^1,2^. The transfer of graphene generates impurities and defects that vitiate the mechanical and electrical properties^3^. Another problem is low bonding strength between graphene and different substrates.

Metal-catalysed graphene growth from SiC followed by a simple but controlled annealing step is a useful approach towards the synthesis of large area graphene layer. Among different metals, Ni, Cu and Co effectively catalysed graphene generation. Juang *et al*.^4^ reported graphene formation on Ni surface by the mechanism of carbon dissolving from SiC substrates by rapid heating. Machač *et al*.^5^ demonstrated a feasible route for graphene growth by annealing of Ni/SiC structures, highlighting the role of Ni layer thickness and a shortage of Ni amount in the structure. Surface Ni-silicidation reaction is also an effective mean for preparation of few-layer graphene (FLG) by controlled thermal annealing of SiC crystals, which was successfully demonstrated in Ni/6H-SiC (0001) system, with the best graphene structure obtained at 800 °C^6^. The similar approaches are also valuable for the transfer-free synthesis of graphene on SiO_2_^7^, sapphire and Cu_2_O (111) substrates^2,8^. Besides Ni and Cu, another transition metal, Co, was investigated as an attractive catalyst for transfer-free graphene growth. By deposition of Co layer on SiC, the FLG was grown via selective reaction between Co film and SiC substrate at high temperatures followed by the rapid cooling^9^. The question that arises here is related to control of carbon diffusion process since it affects number and quality of graphene layers; particularly, formation of the top CoO layer is actually critical for the synthesis of a high-quality graphene layer^10^. Having in mind these results, a successful synthesis of transfer-free graphene layer by the metal-silicidation approach requires several steps, including deposition of selected metal layer/metal oxide layer, control of processing parameters and carbon diffusion process as well.

Cemented carbides are extensively used materials in space drilling applications for providing a first-hand insight into the geological structure and mineral distribution of planets but also as cutting tool materials^11,12^. Development of a suitable material on the surface of cemented carbide tools could enable acquisition of drilling information, detection and automated correction of certain failure modes, such as auger choking, auger jamming, and bit wear^13–15^. Among different materials, graphene-based composites due to their remarkable mechanical, electrical, piezo-resistive and other physical properties become the best option regarding the inherent conventional sensor defects, such as low sensitivity, short service life, and rapid attenuation^16–19^. Especially three-dimensional (3D) graphene with hollow spheres on the surface shows enhanced sensing performances due to large surface area that provides more binding sites, which has great application potential^20^. However, there are no acceptable methods to synthesise in-situ a graphene layer on the cemented carbide surface, especially in regards to following aspects: (1) using readily available a-SiC instead of costly SiC single-crystal as carbon source, (2) the impact of annealing temperature on the number of produced graphene layers and (3) the understanding of Si atoms role in graphene growth and change in the number of graphene layers. If the mechanism of Si acting is not identified, the graphene layercannot be directly grown from a-SiC.

In this study, we propose a novel approach towards the in-situ synthesis of graphene layer on cemented carbides by utilisation of Co as a binding phase in cemented carbides to catalyse the in-situ growth of the graphene film. Herein, Co binding phase catalyses graphitization of amorphous SiC (a-SiC), which is deposited on the cemented carbide at low temperature by the magnetron sputtering technique. In addition, this does not change the carbide performance due to a trace amount of Co involved^21,22^. The clear advantage of this study is an elegant approach towards utilisation of currently existing engineering material and scientific data to develop a sustainable new aspect of cemented carbides without any need of depositing metal oxide layer to control carbon diffusion. The graphene layer with graphene balls on top is formed in-situ by simple and controlled annealing. An extensive physicochemical characterization, including structural, morphological and chemical aspects, was performed to reveal the influence of temperature on the number of formed graphene layers and mechanism of Si atom acting during the graphene matrix generation. This paper opens new horizons towards further enhanced industrial applications of the cemented carbides.

## Results and Discussion

Graphene sheets with graphene balls on top were grown directly on the cemented carbide surface by a new approach that uses Co as a binding phase in the cemented carbides to catalyse the formation of graphene from a-SiC. Moreover, the exact mechanism of graphene formation by annealing a-SiC was revealed. The influence of annealing temperature on morphology, film composition, structural changes and number of graphene layers was systematically studied. Furthermore, the role of Si atoms in graphene formation was discussed regarding the phase transformations and the number of graphene layers.

### Transfer-free graphene growth on cemented carbide by annealing of the a-SiC

**Figure 1:**
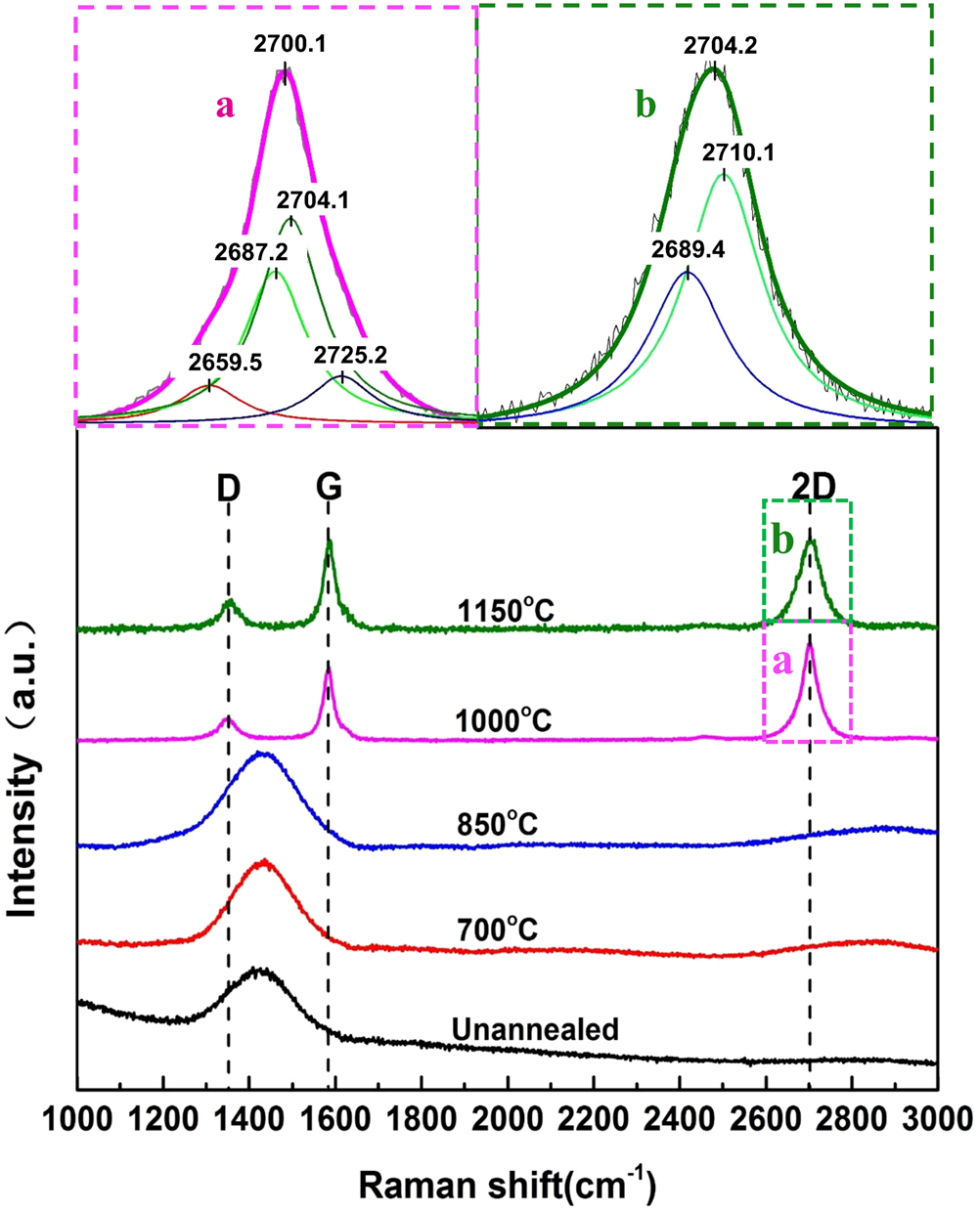
Raman spectra of Co-catalysed a-SiC films at five temperatures. Inset 2-a (top-left corner) is enlarged 2D peak obtained at 1000 °C, and inset 2-b (top-right corner) is the enlarged 2D peak obtained at 1150 °C.

In the present study, Co from cemented carbides is used to in-situ catalyse graphene formation from magnetron-sputtered a-SiC films by an annealing process. The Raman spectra of the deposited a-SiC films annealed at five different temperatures are shown in Fig. 1. Without annealing and at annealing temperatures of 700 and 850 °C appeared diffuse peaks, typical for amorphous structures, which shows that within this temperature range crystallisation of the a-SiC film is not prominent, and the film retains its amorphous structure. When the annealing temperature was increased to 1000 and 1150 °C, D, G and symmetric 2D peaks appeared in the Raman spectra. The G peak indicates the presence of graphite phase, the D peak shows the chaos level of phase structure and the double-resonance 2D peaks suggest that graphene phase was generated^23^.

Data of the D, G and 2D peaks of the graphene obtained at 1000 and 1150 °C are listed in Table 1. Raman spectroscopy can clearly distinguish the number of graphene layers from a single layer to five layers and indicates the defects in graphene structure. The main indicators are intensity ratio of 2D and G peaks (I_2D_/I_G_), full width at half maximum (FWHM) of the peak and the number of fitted Lorentzian peaks^24^. The general equation for determination of the crystallite size, *L*_*a*_ using any laser line in the visible range can be written as^25^:

1 $${L}_{a}({\rm{nm}})=2.4\times {10}^{-10}\lambda {({I}_{D}/{I}_{G})}^{-1}$$

where *λ* is 532 nm. The Raman spectrum at annealing temperature of 1000 °C showed that I_2D_/I_G_ value was higher than 1, FWHM was 45 cm^−1^ and the 2D peak was at 2700 cm^−1^. For a single layer, the intensity of the 2D peak was four times higher than that of the G peak, whilst the intensity of the 2D peak was 1 to 2 times higher than that of the G peak for two layers^26^; FWHM of the 2D peak for a single layer of graphene is in the range of 30–45 cm^−1^, for two layers is in the range of 45–60 cm^−1^ and for three layers is in the range of 65–70 cm^−1^, whilst the FWHM of the 2D peak of graphite was lower than 30 cm^−1 ^^26,27^. Inset 2-a indicates that 2D peak may be deconvoluted into four peaks with maxima at 2659, 2687, 2704 and 2725 cm^−1^, respectively. In general, a 2D peak of a single layer of graphene can be fitted by a single Lorentzian profile, a 2D peak of two layers of graphene can be fitted by four Lorentzian profiles, and a 2D peak of three layers of graphene can be fitted into six Lorentzian profiles, whilst a 2D peak of four or more graphene layers can be fitted only with two Lorentzian profiles^23^. Therefore, it can be concluded that by annealing at 1000 °C, the quality graphene with 2 layers was successfully generated on the cemented carbide.

**Table 1:** Peak positions, peak intensity ratios and graphene grain sizes.

T(°C)	D band		G band		2D band		I_2D_/I_G_	I_D_/I_G_	La(nm)
	Position (cm^−1^)	FWHM (cm^−1^)	Position (cm^−1^)	FWHM (cm^−1^)	Position (cm^−1^)	FWHM (cm^−1^)			
1000	1353	45	1585	31	2700	44	1.304	0.339	56.710
1150	1357	46	1583	34	2704	72	0.964	0.407	47.235

The intensity ratio of the 2D and G peaks at annealing temperature of 1150 °C is lower than that at 1000 °C, whilst the FWHM of the former is greater than of the latter; thus, it is clear that graphene is generated, but the number of layers produced at 1150 °C is obviously higher. The 2D peak may be fitted by two Lorentzian profiles with maxima at 2689 and 2710 cm^−1^ (Inset 2-b), respectively. It is suggested that the number of graphene layers was increased. In addition, the crystallite size of graphene (*L*_*a*_) decreased and the I_D_/I_G_ ratio increased, indicating that the number of film defects increased with the annealing temperature^7,28^. The reason for the increase of a number of defects with temperature is the temperature increase leads to the increase in the number of graphene layers. This induces the formation of boundaries among the graphene layers and the increase of the ratio of I_D_/I_G_.

**Figure 2:**
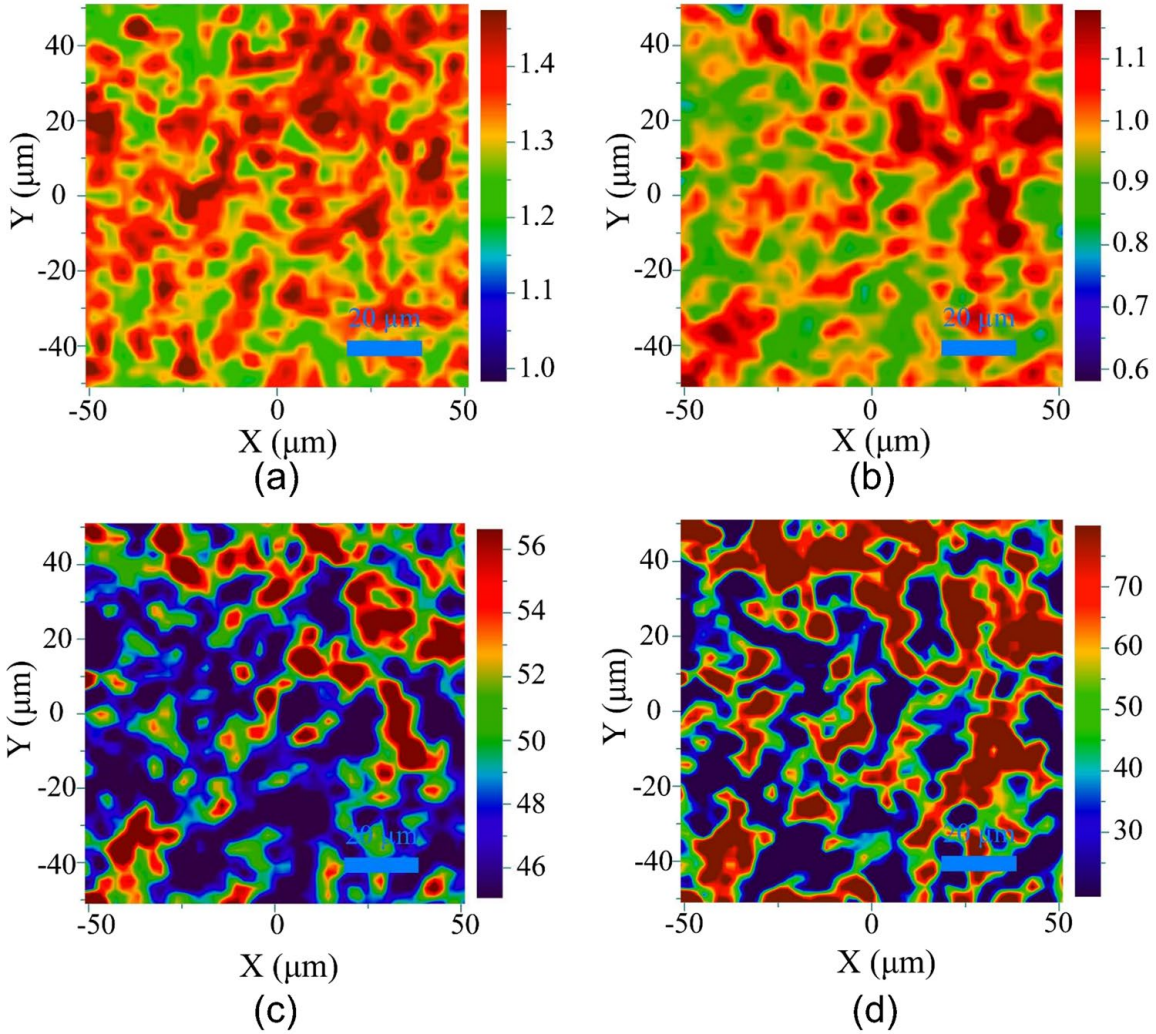
Raman mapping of the graphene film grown on the cemented carbide surface by annealing of a-SiC (size of 100 × 100 *μ*m^2^: (**a**) I_2D_/I_G_ peak ratio at 1000 °C; (**b**) I_2D_/I_G_ peak ratio at 1150 °C; (**c**) FWHM of 2D peak at 1000 °C; (**d**) FWHM of 2D peak at 1150 °C.

Figure 2 shows I_2D_/I_G_ ratio and FWHM of Raman mapping of the graphene film synthesised at two annealing temperatures, 1000 and 1150 °C, with the size of 100 × 100 *μ*m^2^. As shown in Fig. 2a, at 1000 °C I_2D_/I_G_ ratio is in the range of 1.1–1.5, and FWHM, Fig. 2c, in the range of 45–57 cm^−1^. At 1150 °C, Fig. 2b, I_2D_/I_G_ ratio value drops and ranges from 0.7 to 1.2, whilst FWHM value, Fig. 2d, ranges partly from 65 to 80 cm^−1^ but also less than 30 cm^−1^. From these findings can be seen that the graphene film has high quality and consists of 2 layers at 1000 °C, whilst at 1150 °C, the film consists of several layers of graphene and graphite phases.

Thus, in the suitable annealing temperature range (1000–1150 °C), the graphene film was formed on the cemented carbide by Co-catalysed a-SiC transformation. When the annealing temperature was increased from 1000 to 1150 °C, the number of graphene layers, as well as the number defects of the graphene films, were increased, and the quality of the graphene layer was degraded.

### Influence of the annealing temperature on the number of graphene layers

The number of graphene layers is an important parameter to distinguish between graphene and graphite phases and to gauge the quality of graphene. Therefore, it was attempted to investigate how the annealing temperature influences the number of graphene layers. The following approaches were applied: (1) probing the impact of the annealing temperature on the graphene layer structure by XRD; (2) SEM monitoring of morphological changes of the graphene layer upon the annealing process; and (3) analysis of the annealing temperature impact on the graphene film composition by EDS.

**Figure 3:**
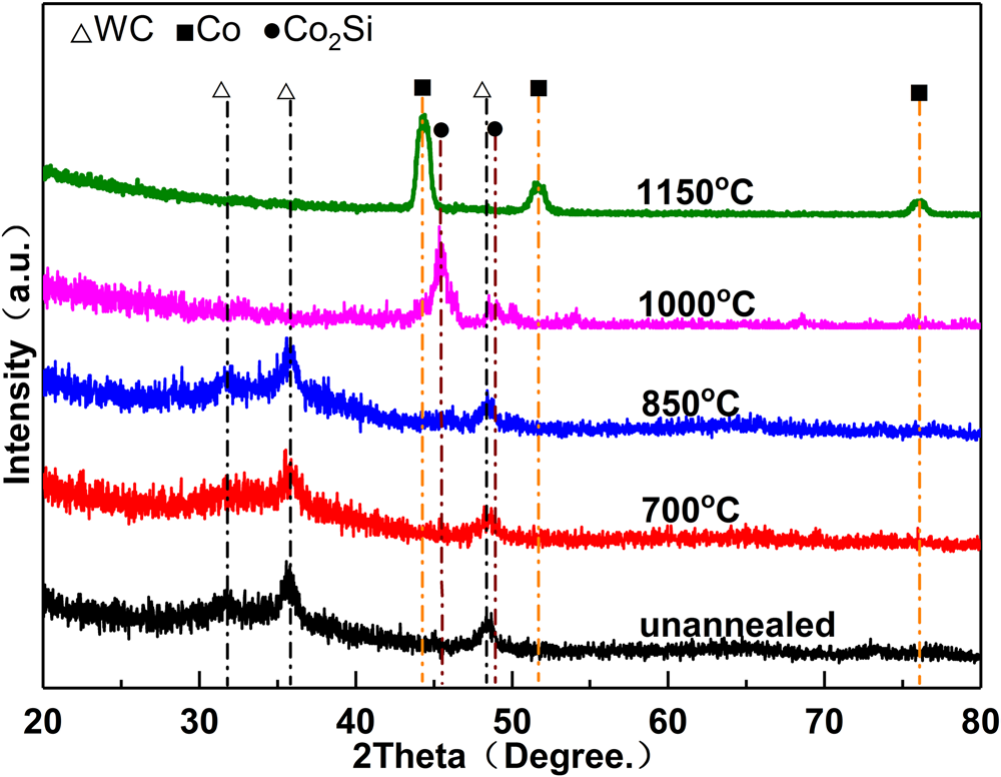
XRD patterns of a-SiC films at five annealing temperatures.

**Figure 4:**
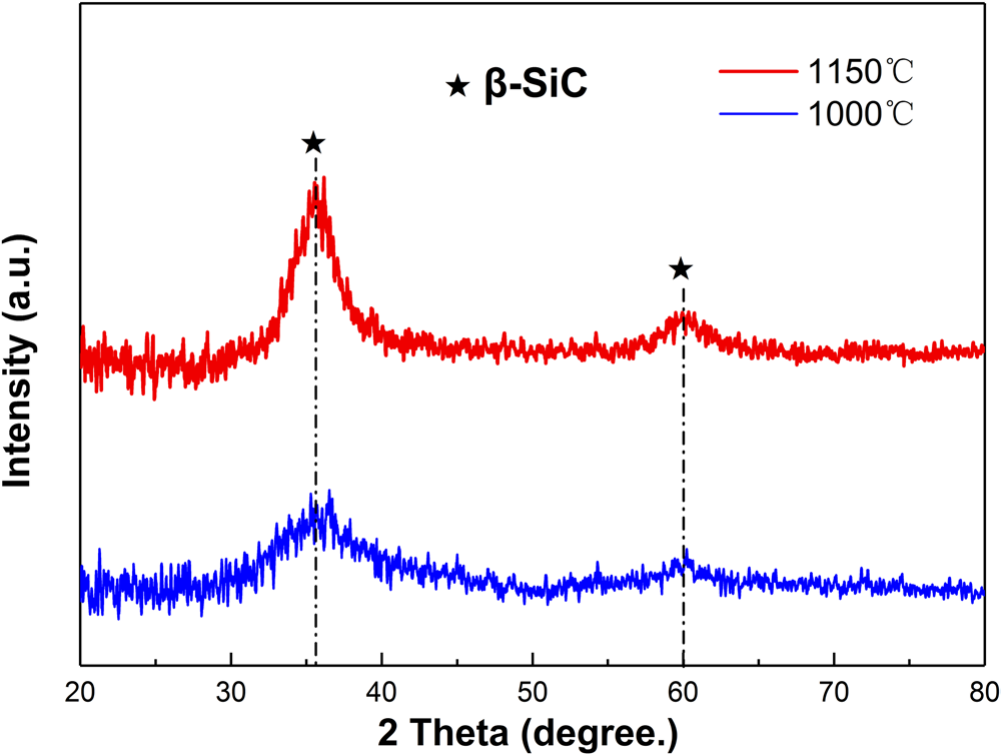
XRD patterns of a-SiC films on the surface of Si slices at annealing temperatures of 1000 and 1150 °C.

Figure 3 shows the XRD patterns of a-SiC films precipitated on cemented carbide after annealing at five different temperatures. The results of XRD analyses show that only the basal peak (WC) appeared without annealing and even after annealing at 700 or 850 °C. These peaks are not very sharp below 850 °C because of the effect of a-SiC on the surface. This indicates the selected temperatures are inadequate for a-SiC film crystallisation. The results are consistent with the results of Raman spectroscopy. When the annealing temperature was increased to 1000 and 1150 °C, the phases of the Co_2_Si and Co, respectively, appeared in the XRD spectra, denoting that within this temperature range Co diffused from cemented carbide into the SiC film and reacted with SiC, thus preventing the crystallisation of a-SiC. However, the diffraction peak of β-SiC appeared on the surface of the Si slice at 1000 and 1150 °C, (Fig. 4), indicating that the a-SiC film was definitely engaged in the crystallisation process, and, thus, confirming the beneficial role of Co.

Different products were generated at different annealing temperatures by the catalytic reaction of Co and a-SiC. The structure of precipitated graphene layer was also changed with temperature. Systematic research on the impact of the annealing temperature on the structure of precipitated graphene layer could assist in revealing the mechanism by which the temperature causes the change in the number of graphene layers. Upon heating, Co and SiC interact, generating the Co-Si compound saturated with C atoms^3,6^. The Gibbs free energy of the Co-Si compound is lower than that of SiC, so the reaction (Co + SiC → Co_x_Si_y_ + C) is a thermodynamically spontaneous reaction. If the reaction temperature is higher than 600 °C, Co_2_Si is generated (2Co + SiC → Co_2_Si + C), and if the temperature is higher than 800 °C, the materials further react to form CoSi (Co_2_Si + SiC → 2CoSi + C)^29^. The formation of graphene is based on the reaction mechanism where C is isolated on the surface of the Co/Co_x_Si_y_ film, owing to the decrease in solubility of C with temperature decrease^30^. As for the annealing temperature of 1000 °C, the Raman analysis shows that graphene was synthesised from the a-SiC film, which is the process catalysed by Co. Because the number of graphene layers was low, the graphite phase was not detected by XRD at approximately 26.5 ° (2θ). The Co reacts with the a-SiC film to form Co_2_Si, and the Co/Si atomic ratio after reaction may contribute to this reaction product^31^. When the Co/Si atom ratio increases, a compound phase in the Co-Si bi-component system approaches Co, and the CoSi compound transforms to Co_2_Si^28^. Thus, at the annealing temperature of 1000 °C, Co sufficiently reacts with the a-SiC film to catalyse graphene generation, generating carbon-saturated Co_2_Si.

Unlike that at 1000 °C, Co reacts with a-SiC at 1150 °C, and there is only the Co phase without Co_2_Si compound. The same as afore-mentioned, Co reacts with the a-SiC film at 800 °C to preferentially generate CoSi. At the annealing temperature of 1150 °C, the CoSi compound in the film disappeared, and the Co phase appeared. Meanwhile, the number of the generated graphene layers increased and the graphite phase could be also detected.

Therefore, in the suitable annealing temperature range (1000–1150 °C), Co diffuses in a-SiC film, catalysing graphene formation. At 1000 °C, Co reacts with a-SiC to form the Co_2_Si compound saturated with C atoms, and the quality graphene layer with low I_D_/I_G_ (0.339) and high I_2D_/I_G_ (1.304) ratio is obtained. When the annealing temperature is further increased, the Co_2_Si compound transforms into Co, and the amount of precipitated C is increased, as well as the number of graphene layers.

**Figure 5:**
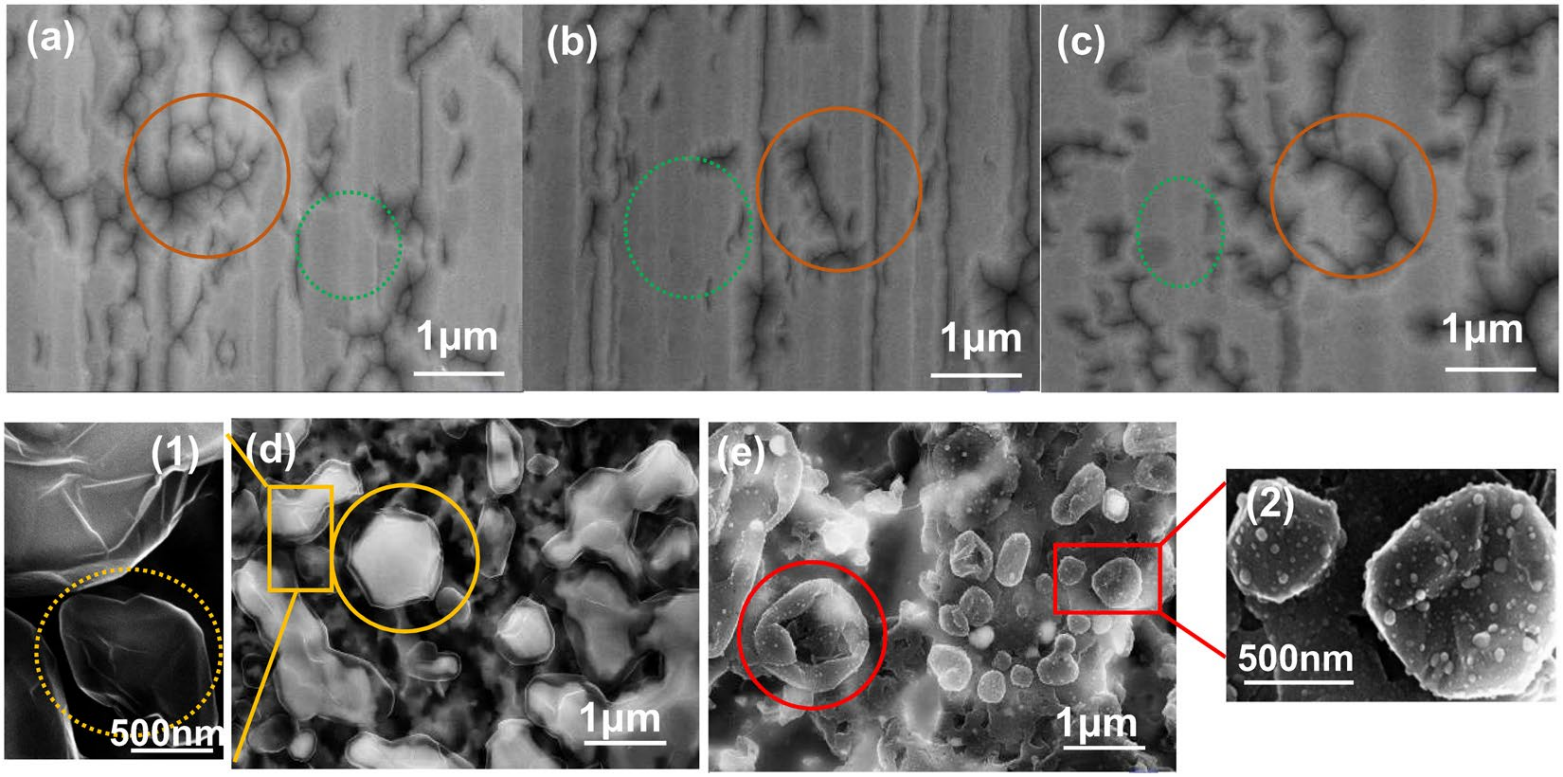
SEM micrographs of the a-SiC films annealed at five temperatures; (**a**) not annealed; (**b**) annealed at 700 °C; (**c**) annealed at 850 °C; (**d**) annealed at 1000 °C; and (**e**) annealed at 1150 °C. Inset 5–1 (on the left) is the enlarged area obtained at 1000 °C, and inset 5–2 is the same for the micrograph obtained at 1150 °C.

The impact of the annealing temperature on graphene morphology is shown in Fig. 5. The dendritic defects and large block regions generated during deposition remain in the a-SiC film even at temperatures of 700 or 850 °C indicating the insufficient atomic activity. At 1000 °C, white particles covered by a transparent graphene film appear on the a-SiC film surface, and the morphology of graphene films is wrapped around the particles with 3D spheres (Fig. 5d). The magnified area in the yellow circle reveals the morphology and particle size of the transparent graphene film, and typical graphene film wrinkles can be observed (as shown in Inset 5–1). If white particles disappear, the transparent graphene film forms hollow graphene balls (as shown by the yellow dotted line in inset 5–1). Compared with the SEM micrograph at 1000 °C, the white particles almost completely disappear at 1150 °C, the hollow graphene balls are formed (as shown by the red circle in Fig. 5e), whilst the transparency of the film is relatively poor, implying the increased thickness of the graphene film (Fig. 5e). Moreover, the wrinkles on the surface of the graphene balls are deeper and the transparent reduction so the film of the hollow graphene balls becomes thicker (as shown in Inset 5–2).

The presented results show that at annealing temperatures of 1000 and 1150 °C, Co diffused into a-SiC film and catalysed the formation of graphene thereon. The metal reacted with a-SiC film and metal silicide spontaneously agglomerated to reduce the surface energy, which is in agreement with previously reported results that the graphene film is generated preferentially at areas where the metal disappeared after contraction^32^.

Thus, it can be concluded that at the annealing temperature of 1000 °C, the graphene balls were precipitated from Co_2_Si particles agglomerated on cemented carbide; hollow graphene balls were generated and graphene sheets with graphene balls were obtained. When the annealing temperature was increased to 1150 °C, Co_2_Si particles disappeared, the film of hollow graphene balls became thicker and the number of graphene layers increased. Such graphene sheets, with hollow graphene balls that meet the requirement of the high specific surface area for an ideal graphene film, can be beneficial for improvement of the efficiency of strain sensors by the reduction of size and power consumption^23^.

**Figure 6:**
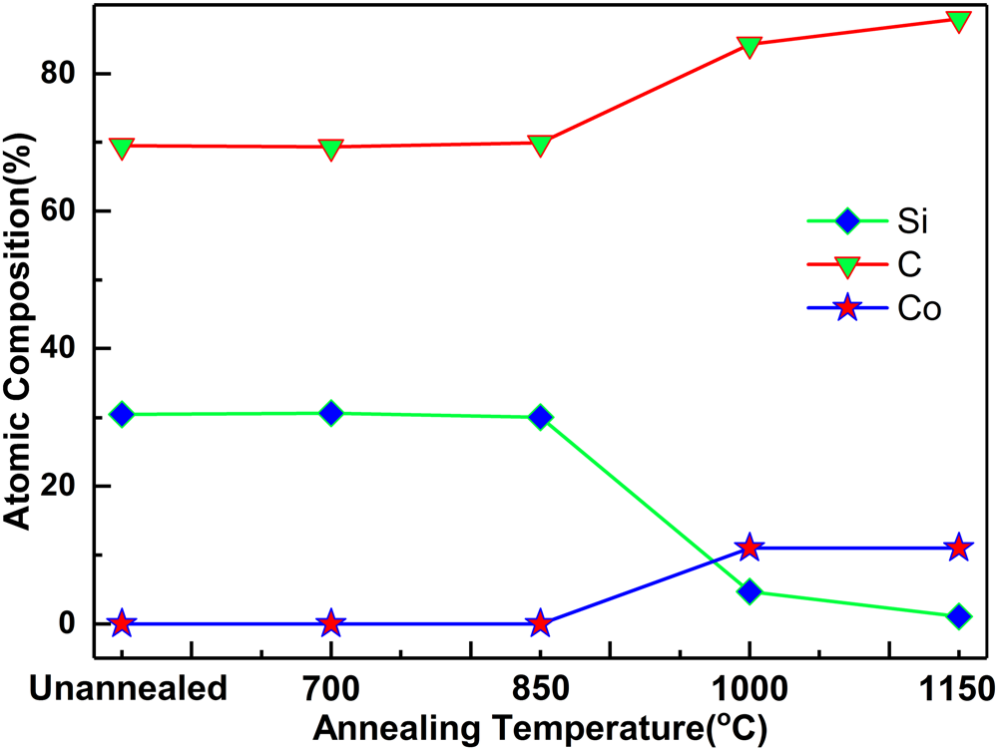
EDS results of a-SiC films annealed at five different temperatures.

Influence of annealing temperature on the chemical composition of graphene film was monitored by EDS. In the temperature range of 1000–1150 °C, when the annealing temperature was increased, the Co_2_Si compound disappeared, so the Co phase appeared in the film, and the number of generated graphene layers increased. In the Co-Si system, different Co/Si ratios resulted in different Co-Si phases^29^. The mechanism by which the precipitation layer structure of segregated C was altered with temperature may be revealed only by a systematic research of the impact of the annealing temperature on the atomic content during the synthesis of graphene. Figure 6 shows the EDS results of the a-SiC film at five annealing temperatures. The contents of Si and C remain essentially unchanged, and Co particles did not exist up to 850 °C (Fig. 6). This indicates that in the temperature range of 700–850 °C, Co binding phase in cemented carbide is unable to diffuse into the a-SiC film. The Co can only react with Si to generate Co_2_Si and the reaction with SiC is not possible, so it does not appear in the a-SiC film. When the annealing temperature was increased, Co atoms appear in the film, the content of Si atoms decreases and the content of C atoms becomes higher, which implies that Co diffused into the a-SiC film.

Unlike conventional graphene epitaxial growth by SiC that requires high temperatures (>1200 °C) and high vacuum (10^–4^ Pa) conditions for Si sublimation from SiC film to enable restructuring of C atoms and graphene formation, graphene can be synthesised at low temperature using a SiC film with Co catalyst^33^. This study shows that at 1000 °C, Co diffused into the a-SiC film and at Co concentration of 11.43%, the content of Si dropped from 30.5% to 4.22%, whilst the amount C atoms increased to 84.35%. It suggests that Co catalysed the sublimation of Si and the restructuring of C atoms, decreasing the content of Si and rearranging C atoms to generate graphene. Compared to that, at 1150 °C the content of Co atoms remained essentially unchanged, 10.83%, the amount of Si was even lower, 0.93% and the amount of C atoms increased to 88.24%. Having in mind the afore-mentioned results of Raman and XRD measurements, it can be stated that at the annealing temperature of 1150 °C, the Co-catalysed sublimation of Si was accelerated, inducing Co_2_Si cracking and the increase in a number of segregated carbon atoms in Co layer, as well as the number of graphene layers.

### Action mechanism of Si atoms in Co-catalysed graphene generation

At 1000 °C, the sublimation of Si atoms was accelerated by Co catalysis, so the content of Si in the film decreased, and C was segregated to generate graphene. When the annealing temperature was increased to 1150 °C, the Co-catalysed sublimation of Si atoms was enhanced, decreasing the content of Si, which induced Co_2_Si compound disappearance and the increase in a number of formed graphene layers. Therefore, the change of Si content in the film has a direct impact on graphene generation and the number of layers. Thus, the role of Si atoms is very important for proper understanding of Co-catalysed graphene formation from a-SiC and the change in the number of graphene layers as well.

**Figure 7:**
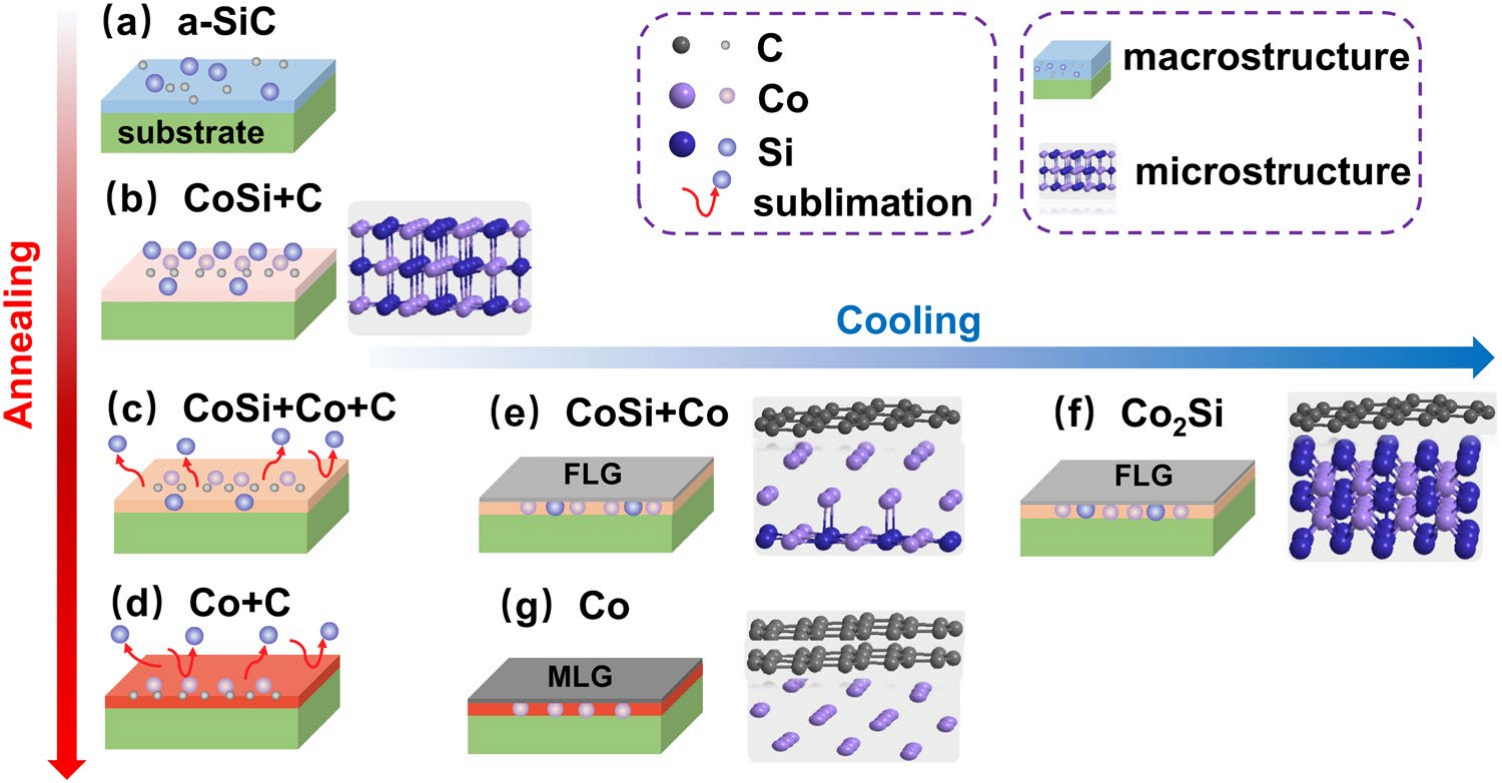
The schematic illustration of graphene formation mechanism by annealing of a-SiC film on the surface of cemented carbide: the changing trend of temperature is demonstrated by the arrow; colours: from shallow to deep means that temperature changes from low to high for annealing and from high to low for cooling.

Figure 7 shows the proposed mechanism describing the role of Si atoms in both Co-catalysed graphene generation from a-SiC film and change in the number of layers. The microstructure and changes in the film are demonstrated by the ball-and-stick structure. A variation in the content of Si atoms in the film may alter the structure of Co metal and causes the change in graphene formation and the number of graphene layers. Three different parts of Si atoms behaviour can be distinguished: (a) Si reacts with Co and C particles are freed (blue represents Si particles in the film, purple represents Co particles in the film and grey represents C particles, Fig. 7b); (b) the content of Si particles decreases and C particles precipitate to generate graphene (C particles are combined to generate the FLG, as shown in Fig. 7e) and (c) Si particles disappear and the number of graphene layers increases (C particles (gray) clusters are gathered to generate the MLG, as shown in Fig. 7g).

The proposed three-step mechanism corroborates well with the experimental findings.

1. **Si reacted with Co to generate the compound saturated with C phase**. During annealing, Co diffused into and reacted with a-SiC film (Fig. 7a), particularly with Si particles to generate CoSi, and free C was distributed in the product (Fig. 7b). XRD results in Fig. 4 illustrate that if the annealing temperature is higher than 850 °C, Co reacts with the a-SiC film to generate the C-saturated CoSi compound.
2. **If the content of Si atoms decreases**, **C atoms segregate in Co layer to generate graphene**. As shown in Fig. 7c–g, the Si content in the film decreased as Si atoms sublimated, and the CoSi compound cracked to generate free Co metal (Fig. 7c,d). As the temperature decreased (during cooling), the solubility of C in the Co metal phase would decline and C atoms in the Co layer would precipitate to form graphene (Fig. 7e,g). The analysis of Raman (Fig. 1) and EDS spectra (Fig. 6) showed that when the annealing temperature was 1000 °C, Co-catalysed Si sublimation and transport of C atoms through free Co metal to generate the graphene layer.
3. **If Si particles disappear**, **the Co-Si compound is not generated**, **and the number of graphene layers increases**. As shown in Fig. 7e, during cooling, C particles passed through Co layer and precipitated to generate graphene; remnant Si particles reacted with Co to generate Co_2_Si and free Co phase disappeared, so C particles stopped precipitating and forming graphene, producing only a few graphene layers (Fig. 7f). When the annealing temperature was increased, Si atoms in the film disappeared (Fig. 7d); during cooling, Co metal was not engaged in the reaction, the Co_2_Si compound was not generated, so the precipitation of C atoms was actually enhanced and the number of graphene layers was increased (Fig. 7g). EDS results in Fig. 6 show that with further increase in the annealing temperature, the Si content in the film significantly decreased and almost disappeared below the detection limit; at the annealing temperature of 1000 °C, the Co_2_Si compound disappeared, and only the free Co layer existed in the film, whilst the number of graphene layers increased.

Summarising the afore-mentioned findings, the influence of Si particles is a synergistic effect of three different actions: Si particles sublimation, CoSi compound cracking and C atoms precipitation through a free metal layer to generate graphene. When the content of Si in the film decreased, the Co_2_Si compound formed, the free metal layer disappeared and the FLG was generated. When Si particles disappeared, the Co-Si compound was not generated, so the amount of free Co phase increased, as well as the number of graphene layers. The content of Si determined the changes in the Co phase and influenced the generation of graphene and the change in the number of graphene layers.

## Conclusions

This study shows an elegant approach for a novel synthesis of high-quality graphene on the surface of cemented carbides. It sublimes current understanding of metal-catalysed transfer-free graphene growth and highly applicable engineering material as cemented carbide. Without any need for prior deposition of metal/metal oxide layer, this approach offers a feasible route to produce transferable graphene film, providing the explanation of the underlying chemical mechanism.

Co, as a binding phase of cemented carbides, was successfully used to catalyse a direct formation of graphene film with graphene balls on top from the a-SiC film on the cemented carbide. The influence of the annealing temperature on structure and morphology of the generated graphene film, change in atomic composition and the number of generated graphene layers were systematically investigated. The mechanism that describes the role of Si atoms was revealed based on the relationship between the change of Si atomic content and the generation and change in the number of graphene layers.

The three most important findings of this study can be summarised as follows: (1) graphene is directly generated from the a-SiC film on the surface of cemented carbide catalysed by Co binding phase via the annealing process in the temperature range of 1000–1150 °C. When the annealing temperature is increased (1150 °C), the number of graphene layers increases and the graphene quality becomes poorer. (2) Influence of the annealing temperature on the number of graphene layers is related to changes in the film composition and structure. In the suitable temperature range (1000–1150 °C), Co catalyses Si sublimation, and C particles segregate to generate graphene. The related changes in atomic and phase compositions at the higher annealing temperature induced the increase in the number of graphene layers. (3) The content of Si influences the Co pattern and causes the generation of graphene and change in the number of graphene layers. The mechanism of Si atoms acting actually includes three subsequent parts: (a) when the content of Si particles decreases, C particles segregate in the Co layer to generate graphene; (b) Si particles react with Co to generate Si-Co compound, so the free Co layer disappears, and the number of precipitated graphene layers is low; and, (c) when Si particles disappear, no reaction with free Co metal occurs, and the number of graphene layers increases.

This research study provides a new deep insight into the transfer-free direct growth of graphene on the surface of cemented carbides, explaining the mechanism of chemical processes behind, especially underlying the structural, chemical and morphological aspects of the graphene layer formation.

## Methods

### Preparation of the graphene layer

The a-SiC films were deposited by a mid-frequency magnetron sputtering technique. There were two kinds of substrate materials: monocrystal Si wafer was used for control analysis of the structure, whilst cemented carbide YG 8 (WC-Co 8%) was used for the analysis of the graphene structure. The sample preparation method included following steps: (1) the substrate was cleaned in both acetone and absolute ethanol by ultrasonication in each medium for 10 min, and (2) the substrate was dried by nitrogen and placed in a vacuum chamber for deposition. The reaction of Si target and acetylene, which provided the source of silicon and carbon atoms, respectively, was used for the a-SiC film deposition. The main experimental parameters include basic pressure (3.0 × 10^−3^ Pa), Ar ion bombardment duration (20 min), bias (−800 V), deposition pressure (0.5 Pa), C_2_H_2_ flow rate (35 SCCM), substrate bias (−100 V), target current (22 A) and deposition temperature (150 °C). The film thickness was approximately 2.0 μm.

**Figure 8:**
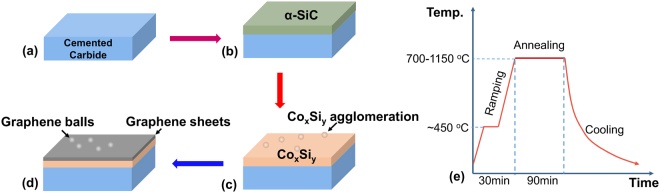
Schematic illustration of the graphene matrix growth process: (**a**) The substrate of cemented carbide. (**b**) a-SiC sputtered on the substrate to produce carbon source. (**c**) Heating in a vacuum high-temperature furnace. (**d**) After cooling, C precipitates and forms the graphene sheets and balls. (**e**) Annealing process.

The a-SiC coating films deposited on both kinds of substrates were shortly isothermally treated at 450 °C in a vacuum high-temperature furnace. Subsequently, the coated films were heated and annealed in the vacuum furnace at annealing temperatures of 700, 850, 1000 and 1150 °C for 90 min, and the pressure in the furnace was maintained below 5 × 10^−4^ Pa. The binding phase, Co, of cemented carbide, was used to catalyse the in-situ formation of the graphene matrix from the a-SiC film. After annealing, the test blocks were cooled with the furnace in vacuum. Figure 8 shows the steps required for the growth graphene matrix.

### Characterisation of the synthesised graphene layers

Crystal structure analysis was performed by X-ray diffraction (XRD; Bruker D8 Advance, CuKα, 40 kV, 40 mA, the diffraction angle is 2° (2θ), 2θ range 20–80° and a step size of 0.02°). Valence bond and the number of layers of graphene films were determined by Raman spectroscopy (LabRAM HR Evolution, HOEIBA Jobin Yvon, France) using 532 nm laser excitation at room temperature, and layer uniformity is revealed by Raman mapping; surface morphology was identified by scanning electron microscopy (SEM, JEOL JSM 6301 F), while compositions of the film surfaces were analysed by energy-dispersive X-ray spectrometry (EDS). The atomic composition was obtained by taking the average of six measurements on the surface of the individual sample.
